# The Hypotensive and Vasodilatory Effects Observed in Rats Exposed to *Chiranthodendron pentadactylon* Larreat Flowers Can Be Attributed to Cyanidin 3-*O*-Glucoside

**DOI:** 10.3390/molecules28237698

**Published:** 2023-11-22

**Authors:** Juan Luis Escobar-Ramírez, Jacinto Santiago-Mejía, Maribel Soto-Núñez, Oscar Salvador Barrera-Vázquez, Roberto Vargas-Querea, Gil Alfonso Magos-Guerrero

**Affiliations:** Department of Pharmacology, Faculty of Medicine, University National Autonomous of Mexico, Mexico City 04510, Mexico; re-harakhty@hotmail.com (J.L.E.-R.); samj@unam.mx (J.S.-M.); farma_estrogenos@yahoo.com (M.S.-N.); osbarrera6@gmail.com (O.S.B.-V.); gcomrv@gmail.com (R.V.-Q.)

**Keywords:** *Chiranthodendron pentadactylon*, antihypertensive, vasorelaxant, Wistar rat, cyanidin 3-*O*-glucoside

## Abstract

*Chiranthodendron pentadactylon* Larreat is a tree native to southeastern Mexico and Guatemala. Its flower is used in Mexican folk medicine to treat a variety of diseases, including conditions of blood pressure. However, scientific information on its usefulness in this pathology is lacking. The present study evaluates the effect of a methanolic extract (ME) from the flower and its active constituents on heart rate (HR) and mean arterial pressure (MAP) in anesthetized rats (MAPHR). The study also analyzed the effects on rat-isolated aortic rings (RIAR) and the rat mesenteric arterial bed (MABR). Active fractions were chromatographed, which led to the isolation of cyanidin 3-*O*-glucoside (C3G) identified through HPLC. The *Chiranthodendron pentadactylon* flowers produced hypotensive and vasorelaxant effects associated with C3G. The vasorelaxant effect is a mechanism underlying the synthesis and release of nitric oxide (NO). Neither cholinergic receptors nor prostaglandins are involved. ME and C3G cause cardiovascular depression in anesthetized rats via cholinergic and prostanoid mechanisms. Our research expands the scientific understanding of the flowers on the rat cardiovascular system. This amplifies the appreciation of the flower’s ethnomedicine employed to control blood pressure. However, researchers need to conduct toxicity studies to determine the safety of this plant.

## 1. Introduction

*Chiranthodendron pentadactylon* Larreat is a tree of the Malvaceae family, native to Mexico and Guatemala. The Aztecs named this tree “macpalxochitl”, combining “macpalli” and “Xochitl” to highlight the hand-like shape of its flower [1]. *Chiranthodendron pentadactylon* flowers are recognized as a medicinal plant in the de la Cruz-Badiano Codex. This writing is the oldest medical text in the Americas [2]. *Chiranthodendron pentadactylon* flowers are used medicinally in Mexico for heart disease, cancer, ulcers, ocular pain, headaches, and inflammation. The authors of [3,4,5] were the first to publish the antecedent linking the sale of “flor de manita” in the markets of Mexico City to heart problems. The flower’s employment in controlling blood pressure was first reported by Espinoza [6]. Its use as a remedy for systemic arterial hypertension has been extended [7,8,9]. However, there is a lack of scientific evidence on the cardiovascular effects of this plant and even less on its activity in systemic blood pressure. The present study examines the effects of ME from the fresh flowers of *Chiranthodendron pentadactylon* on the MAPHR and RIAR. In addition, we will isolate the active principle(s) responsible for the hypotensive activity. All this is to support the use of the flowers of this tree in the traditional medicine of Mexico and Guatemala.

## 2. Results

### 2.1. Identification and Quantification of Anthocyanin

**Figure 1:**
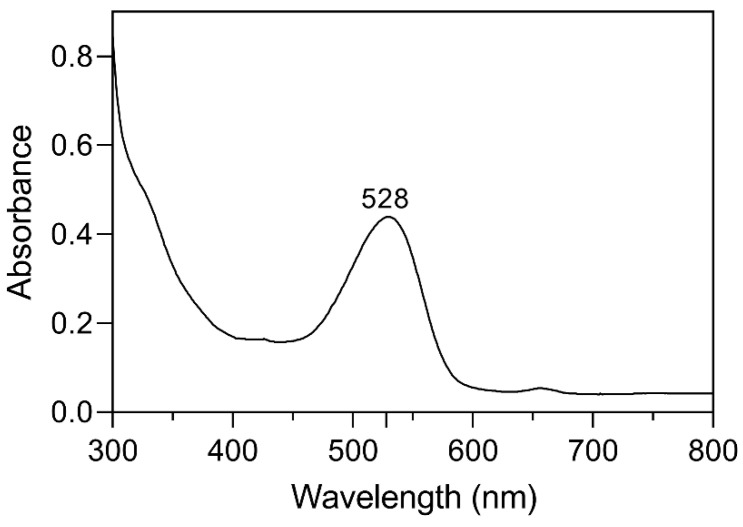
The UV-Vis spectrum of purified C3G dissolved in acidified methanol (0.01% *v*/*v*) with HCl.

A spectroscopic analysis of anthocyanins was performed because of a positive Rosenheim test [10] result on the active ingredient extracted from ME. The active principle that we separated has the same coloration as the red pigment present in the flowers, previously identified as C3G [11]. The ultraviolet-visible spectrum of the active compounds in the acidified ME extract displayed the highest absorbance at 528 nm (Figure 1). It was previously mentioned by Chandra Singh et al. [12] that C3G has an absorbance of 528 nm in acidified methanol (0.01% *v*/*v*) with HCl.

**Figure 2:**
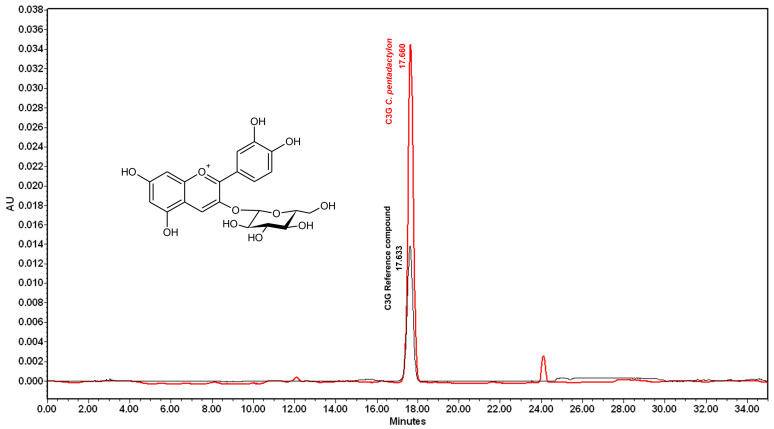
HPLC chromatogram of C3G isolated from *Chiranthodendron pentadactylon* flowers and the C3G standard.

Spectrometric analysis of the HPLC chromatogram of C3G compounds is presented in Figure 2. The C3G from the *Chiranthodendron pentadactylon* flowers had the same time retention as the C3G refence compound (17.633 min and 17.660 min).

**Figure 3:**
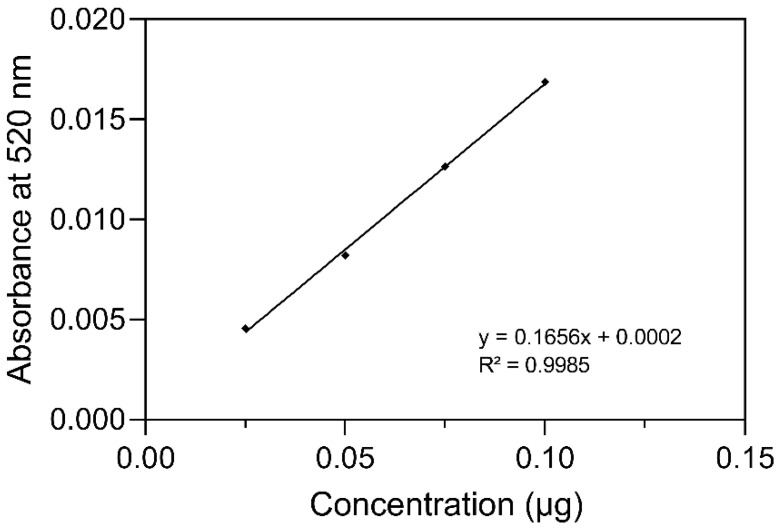
Calibration curve of the C3G standard.

The curve of the C3G standard was linear (R^2^ = 0.9985) in the concentration range of 0.025 to 0.1 µg/mL (Figure 3). The concentration of C3G isolated from the *Chiranthodendron pentadactylon* flowers was estimated at 47 ± 12.6 mg/100 g of fresh weight (FW).

### 2.2. Effects of Increasing Doses of ACh, ME, or C3G on the MAPHR

**Figure 4:**
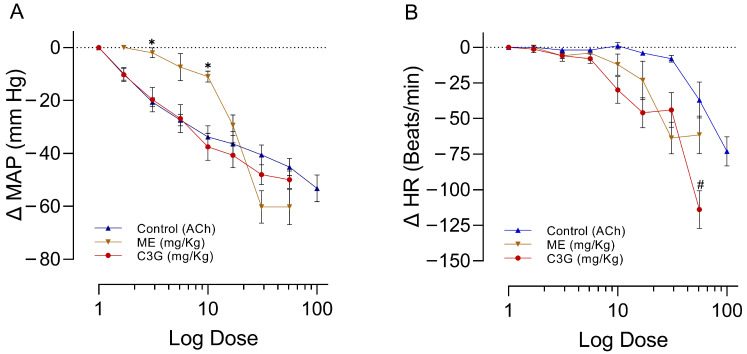
Effect of increased ME, C3G, and ACh doses on the MAP (**A**) and HR (**B**) of rats under anesthesia. ACh shows the functionality of the experimental model’s reactivity. The abscissae correspond to the incremented doses of the treatments, with a sample size of six for each treatment. ACh is quantified in µg, while ME and C3G are measured in mg. The ordinates show changes in MAP or HR. The symbols represent the mean ± SEM (standard error of the mean) of six experiments. ✱ shows the significant difference between the control MAP and ME values, and # shows the significant difference between the control HR and C3G values. *p* < 0.05 ANOVA post hoc Dunnett’s test.

All experiments show similar basal values for MAP and different HRs (*p* < 0.0001) between the control ACh and ME groups (Table 1). Acetylcholine (ACh), ME, and its active ingredient C3G reduced MAP (Figure 4A) and HR (Figure 4B) when administered to anesthetized rats in increasing doses. ACh was used to evaluate the reactivity of the experimental model. As expected, the decrease in MAP and HR produced by ACh is dose-dependent. ACh is the most potent hypotensive agent, employing effective doses of 50 (DE_50_) = 5.9 ± 2.7 µg/kg, followed by C3G (DE_50_ = 5.0 ± 1.8 mg/kg) and ME (DE_50_ = 15.3 ± 2.8 mg/kg). The effect of C3G on the MAP is like that of ACh, while the ME requires higher doses to achieve the maximum ACh effect. At a dosage of 56 mg/kg, C3G displays a greater bradycardic effect than the ACh control (*p* = 0.041). This could be explained if C3G had additional mechanisms of action to stimulate the cholinergic receptor. The ME requires a higher dosage (17 mg/kg) to induce a hypotensive effect, and based on the maximum dose tested, this effect is slightly higher than that of the other two groups (Figure 4A). For more details, please see Tables S1.1 and S1.2 in the Supplementary Data. The supplement presents a comprehensive analysis of all the specific findings, encompassing time, dosage measurements, mean values, standard errors, and *p*-values.

### 2.3. Effects of a Single Dose of ME or C3G on the MAPHR

**Figure 5:**
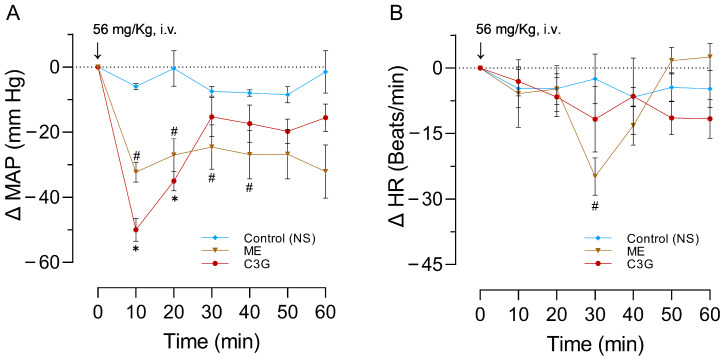
Effects of a single dose of 56 mg/kg of whole ME and its active principle C3G on MAP (**A**) and HR (**B**) of anesthetized rats. The control group receives normal saline (NS). The abscissas correspond to the recorded time of each treatment, with a sample size of six for each treatment, and the ordinates show changes in MAP or HR. The symbols represent the mean ± SEM of six experiments. ✱ shows the significant difference between the control and C3G values, and # shows the significant difference between the control and ME values. *p* < 0.05 ANOVA post hoc Dunnett’s test.

As expected, the experiments started with similar MAP and HR values (Table 2). Figure 5A shows that the intravenous administration of either ME or C3G at a dose of 56 mg/kg in anesthetized rats at 10 min led to a significant reduction in MAP (ME: *p* = 0.032 and C3G: *p* = 0.004). In HR (Figure 5B) at 30 min, the ME produced a significant diminution (*p* = 0.040). The bradycardia, caused by C3G obtained from the flowers, persisted throughout the entire 60 min recording. In contrast, the rats treated with ME had their HR return to its initial value (Figure 5B). For more details, please see Tables S2.1 and S2.2 in the Supplementary Data.

### 2.4. Selection of the Aqueous Extract

**Figure 6:**
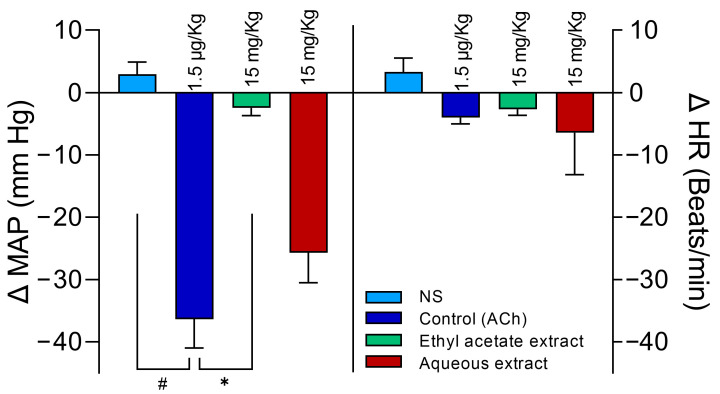
Effect of 15 mg/kg i.v. of the aqueous and ethyl acetate extracts on the MAP and HR of anesthetized rats. Both extracts were obtained from the whole ME of the *Chiranthodendron pentadactylon* flowers. The abscissas correspond to the treatments administered, with a sample size of six for each treatment, and the ordinates show changes in the MAP or HR. The bars represent the mean ± SEM of six experiments. ✱ shows the significant difference between the control MAP and ethyl acetate extract values, and # shows the control versus NS (normal saline) values. *p* < 0.05 ANOVA post hoc Dunnett’s test. In HR, there were no significant differences.

As expected, the experiments started with similar MAP and HR values (Table 3). Anesthetized rats experienced a reduction in MAP that was like that of ACh when administered with an aqueous extraction from the ME at a dose of 15 mg/kg. The same 15 mg/kg administered doses of the ethyl acetate extract did not diminish the MAP when compared to the effects of ACh (*p* < 0.0001). The HR did not show significant modifications in any treatments (Figure 6). For more details, please see Table S3.1 in the Supplementary Data.

### 2.5. Influence of L-NAME, Atropine, and Indomethacin on the Effects of C3G in the MAPHR

**Figure 7:**
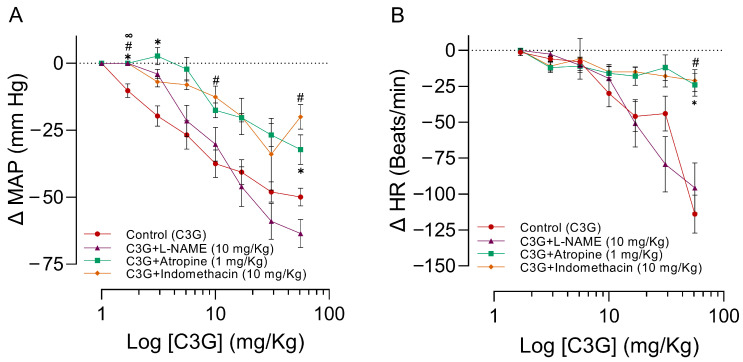
Effects of pretreatments with either atropine, indomethacin, or L-NAME on the changes induced by C3G in the MAP (**A**) and HR (**B**) of anesthetized rats. Abscissae corresponds to increased C3G doses in different pretreatments with a sample size of six for each treatment, and the ordinates show changes in MAP or HR. The symbols represent the mean ± SEM of six experiments. ✱ Shows significant differences between the control and C3G + atropine values, # the control versus C3G + Indomethacin values, and ∞ the control versus C3G + L-NAME values (**A**,**B**). *p* < 0.05 ANOVA post hoc Dunnett’s test.

Table 4 shows the baseline values from MAP and HR of the groups of animals pre-treated with L-NAME, atropine, or indomethacin. The Nω-Nitro-L-arginine methyl ester L-NAME pretreatment significantly increased the MAP (*p* < 0.0001). Intravenous administration of increasing doses of C3G (DE_50_ = 8.6 ± 7.8 mg/kg) in anesthetized rats significantly reduced MAP (Figure 7A) and HR (Figure 7B). The hypotension and bradycardia produced by C3G are dose-dependent. The doses of atropine (3.1, 5.6, and 56 mg/kg) and indomethacin (3.1, 10, and 56 mg/kg) significantly attenuated the effects of C3G over MAP (DE_50_ = 22.4 ± 12.3 mg/kg and DE_50_ = 23.9 ± 7.6 mg/kg), and at doses of 56 mg/kg, both compounds block the HR. The pretreatment with L-NAME attenuates the hypotensive effect of the first two doses of C3G (DE_50_ = 7.6 ±0.8 mg/kg), see Figure 7B. For more details, please see Tables S4.1 and S4.2 in the Supplementary Data.

### 2.6. Effects of Increasing Doses of ME or C3G on RIAR

**Figure 8:**
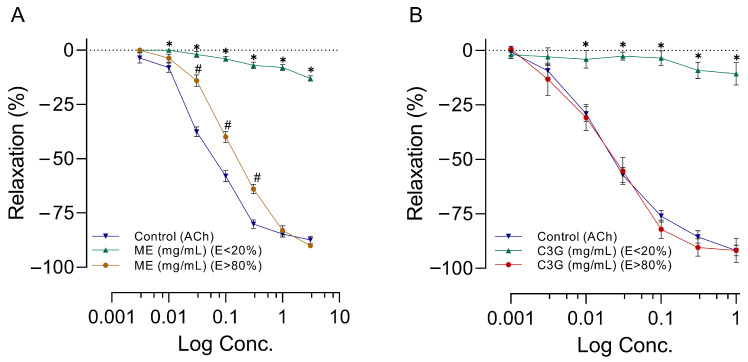
Effects of ME (**A**) or C3G (**B**) extracted from the *Chiranthodendron pentadactylon* flowers on the RIAR system. Abscissae correspond to increased concentration in different pretreatments, with a sample size of eight for each treatment, and the ordinates show relaxation in percent changes. The symbols represent the mean ± SEM of eight experiments. ✱ shows a significant difference between the vasorelaxant effect produced by the control versus ME values with Endothelium (E) < 20%; # shows the control versus ME E > 80% (**A**); and ✱ shows a significant difference between the vasorelaxant effect produced by the control and C3G E < 20% (**B**). *p* < 0.05 ANOVA post hoc Dunnett’s test.

The application of increasing doses of ACh, ME, or C3G to RIAR, which had been previously contracted with norepinephrine (NE = 0.1 µM), led to a dose-dependent relaxation. ME’s vasorelaxant effect at practically all doses depends on the presence of endothelium, as shown by the blockade of this effect when the endothelium is removed (Figure 8A). We observed a similar endothelium-dependent effect on increased doses of C3G (Figure 8B). To achieve maximum vasorelaxant effect, more doses of ME than C3G were required. The concentration that led to 50% inhibition (IC_50_) of the maximum contractile response in experiments realized with endothelium >80% (E > 80%) was IC_50_ = 0.05 ± 0.01 µg/mL for ACh and IC_50_ = 0.14 ± 0.02 mg/mL for ME (Figure 8A). In experiments realized with C3G with endothelium E > 80%, the IC_50_= 0.02 ± 0.01 µg/mL for ACh and IC_50_ = 0.02 ± 0.01 mg/mL for C3G (Figure 8B). As presented in Figure 8, removing vascular endothelium inhibited the concentration-response curves of the ME and C3G. For more details, please see Tables S5.1 and S5.2 in the Supplementary Data.

### 2.7. Influence of L-NAME, Atropine, and Indomethacin on the Effects of ME or C3G of the Flowers on RIAR

**Figure 9:**
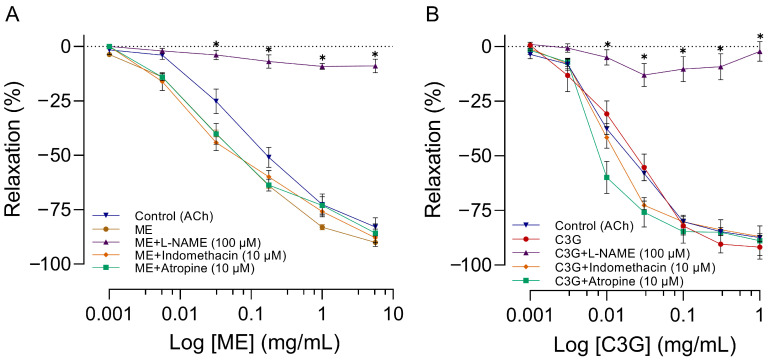
Effects of pretreatments with either atropine, indomethacin, ACh, or L-NAME on the vasorelaxant effect produced by ME (**A**) or C3G (**B**) in the RIAR system. The abscissae correspond to an increased concentration. Ordinates show changes in the vasorelaxant effect caused by ME (**A**) or C3G (**B**) with several pretreatments, with a sample size of eight for each treatment. The symbols represent the mean ± SEM of eight experiments. ✱ shows a significant difference between control and ME + L-NAME values (**A**) and between control and C3G + L-NAME values (**B**). *p* < 0.05 ANOVA post hoc Dunnett’s test.

The vasorelaxation produced by ME (IC_50_ = 0.07 ± 0.01 mg/mL) or C3G (IC_50_ = 0.02 ± 0.01 mg/mL) (Figure 9A,B) on RIAR with endothelium was inhibited by 100 µM L-NAME (an inhibitor of NO synthase) pretreatment. The vasorelaxation induced by ME (IC_50_ = 0.04 ± 0.008) remained unaffected by pretreatment with 10 µM indomethacin (a cyclooxygenase inhibitor) or 1 µM atropine (a muscarinic antagonist). No significant modifications were observed in the concentration-response curves of C3G upon the administration of indomethacin and atropine, as evidenced by IC_50_ values of 0.012 ± 0.01 mg/mL and 0.008 ± 0.005 mg/mL, respectively.

To evaluate the contribution of the endothelium to the vasorelaxant effect of ME or C3G, ACh was used as a positive control (IC_50_ = 0.1 ± 0.02 µg/mL). As expected, ACh produced a significant relaxant effect, which was completely blocked by L-NAME (Figure 9A,B). For more details, please see Tables S6.1 and S6.2 in the Supplementary Data.

### 2.8. Effects of a Single Dose of ME or C3G on the Flowers on the MABR

**Figure 10:**
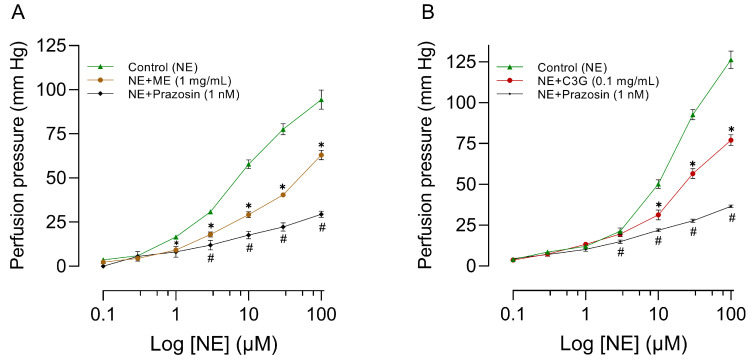
Effect of ME (**A**) or C3G (**B**) on the concentration-response curve produced by NE in mesenteric vascular beds from rats. The abscissae show increases in NE concentration. Ordinates show changes in mm Hg of the perfusion pressure caused by several treatments, with a sample size of six for each treatment. The symbols represent the mean ± SEM of six experiments. ✱ shows a significant difference between the control and NE + ME values and # the control versus NE + prazosin values (**A**); and ✱ control versus NE + C3G values and # control versus NE + prazosin values (**B**). *p* < 0.05 Student’s *t*-test for two related samples.

The effects of ME, C3G, or prazosin on concentration-response curves for norepinephrine in mesenteric vascular beds are illustrated in Figure 10. Incorporating ME (Figure 10A), C3G (Figure 10B), or prazosin in the perfusion fluid results in the antagonism of the concentration-response curve for norepinephrine. Each of the three treatments significantly reduced the maximum perfusion pressure caused by NE. The perfusion pressure, which is directly correlated to vascular resistance, was nearly 20 mm Hg (resting tone). Prazosin was used to evaluate the reactivity of the experimental model. As expected, prazosin antagonized the increase in perfusion pressure produced by norepinephrine. For more details, please see Tables S7.1 and S7.2 in the Supplementary Data.

### 2.9. Influence of L-NAME on the Effects of C3G of the Flowers on the MABR

**Figure 11:**
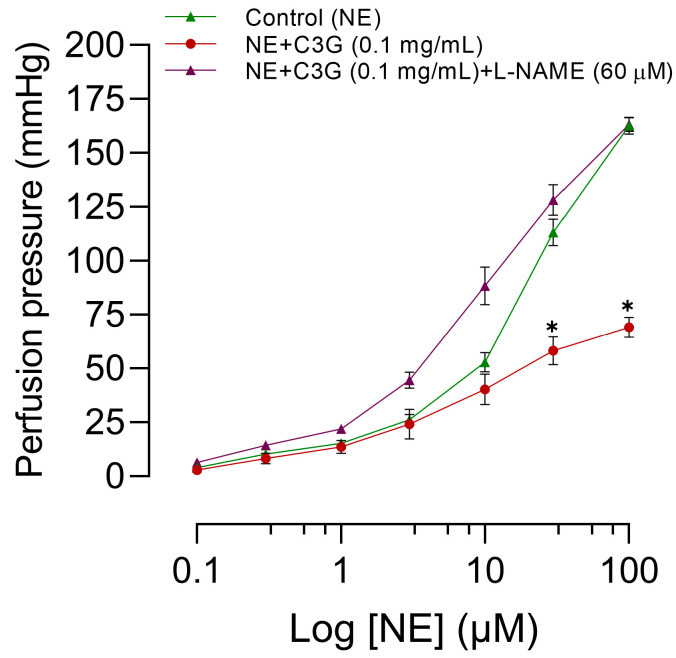
Effect of C3G in the presence or absence of L-NAME on the concentration-response curve produced by NE in mesenteric vascular beds from rats. The abscissae show increases in NE concentration. Ordinates show changes in mm Hg of the perfusion pressure caused by several treatments, with a sample size of eight for each treatment. The symbols represent the mean ± SEM of six experiments. ✱ shows a significant difference between the control and NE + C3G values. *p* < 0.05 Student’s *t*-test for two paired samples.

The effects of C3G with or without L-NAME on concentration-response curves for norepinephrine in mesenteric vascular beds are illustrated in Figure 11. The effect of each concentration was measured in the steady state 5 min after the addition of each dose. Incorporation of C3G into the perfusion fluid results in a rightward shift and flattening of the norepinephrine curve. It significantly antagonizes the increase in norepinephrine perfusion pressure at doses of 31 µM and 100 µM of NE (*p* = 0.004 and *p* < 0.0001, respectively). As expected, C3G and L-NAME in the perfusion fluid increase the perfusion pressure induced by NE. L-NAME prevents the effect of C3G. The perfusion pressure, which is directly correlated to vascular resistance, was nearly 20 mm Hg (resting tone). For more details, please see Tables S8.1 and S7.2 in the Supplementary Data.

## 3. Discussion

The primary contribution of this study is to show, for the first time, that a ME derived from the *Chiranthodendron pentadactylon* flowers produces hypotension and bradycardia in anesthetized rats. In addition, fractionation of ME directed by biological activity results in a single compound that appears to be the responsible principal for the cardiovascular impacts of the flowers. The active fraction of our study exhibits a compound that was previously discovered in the flower by Harborne et al. [11]. Based on an HPLC profile, the compound was identified to be anthocyanin C3G (Figure 2). It is important to note that β-sitosterol, quercetin 3-glucoside, (−)-epicatechin, and tiliroside, among other compounds, have been identified in the flowers of *Chiranthodendron pentadactylon* [11,13,14], which have been linked to reducing blood pressure [15,16,17,18,19,20]. It is reasonable to infer that these active substances may have a role in the cardiovascular effects observed in our results. In contrast to what was expected, the bio-directed fractionation of the ME implemented led to the discovery of a solely active compound. The ME’s solubility and yield could explain why we found only one substance. Because of the high water solubility of C3G, it was concentrated in the aqueous extract, which shows major cardiovascular depression (Figure 6). The flavonoids with lesser polarity are believed to be present in inadequate quantities in the ethyl acetate extract to exhibit substantial effects. It is significant to highlight that, in phytochemical studies of the *Chiranthodendron pentadactylon* flowers, solvents of lower solubility were used for the extraction of substances such as petroleum ether, benzene acetate or chloroform [13], and ethyl acetate [14,21]. In our study, the substantial concentration of C3G obtained from the flowers allowed for testing the aqueous extract using doses of 15 mg/kg in whole rats. The culmination of the purification process of the active fractions resulted in the substance C3G being the major component found.

The anthocyanins belonging to the flavonoid family are found in leaves, stems, roots, flowers, and fruits of diverse plant species as pigments that provide color [22]. C3G, also known as asterin, chrysanthemin, and kuromanin, is a well-known anthocyanin that can be found in significant quantities in several fruits, vegetables, nuts, spices, and beverages [23,24]. The active fraction of our study shows a high concentration of C3G (47 ± 12.6 mg/100 g FW), which implies that the vasodilation and hypotension effects induced by ME in MAPHR, RIAR, and MABR are primarily attributed to this substance. There is an ample amount of literature available to support the extensively reported bioactivity of C3G [25]. To explain the protective effects of C3G against various diseases, including cardiovascular disease, relevant mechanisms such as radical scavenging capacity, epigenetic action, competitive protein-binding, and enzyme inhibition, among others, have been suggested [26].

The prompt appearance of cardiovascular impacts ME, or its active principle C3G, can be explained by the rapid dissemination of C3G or its active metabolites to the tissues, as shown by the pharmacokinetic studies of this substance [27,28]. The rapid penetration of C3G into endothelial cells, facilitated by bilitranslocase [29], and its prompt elimination from plasma (t_1/2_ = 0.36 min) reported by Vanzo et al. [28], may account for the acute hypotensive effect observed with the administration of ME or C3G (Figure 5A). The long-lasting hypotensive effect could also be explained by the formation of active metabolites of C3G [27,28].

Within our study, it was observed that ME, alongside its active principle C3G, resulted in vasorelaxation of large (RIAR) and smaller vessels (MABR), as shown in Figure 8 and Figure 10, respectively. The magnitude of the vasorelaxant effect is determined by concentration and endothelium. Our results agree with the vasorelaxant effect observed by Perrusquia et al. [30] and Ibarra-Alvarado et al. [31] in RIAR, but differ in the IC_50_ reported. In our investigation, the IC_50_ was significantly lower (0.14 mg/mL) compared to the 18 mg/mL reported by Perrusquia et al. [30], and highest compared with the 0.030 mg/mL reported by Ibarra-Alvarado et al. [31]. Our findings show that the active principle C3G, present in crude ME, can exert a similar vasorelaxant effect to ME with a lower concentration of IC_50_ = 0.02 mg/mL. The hypotensive effect observed with the intravenous administration of ME or C3G in the anesthetized rat could be explained by the vasorelaxation produced in the small resistance vessels (MABR). According to previous research conducted by Xu et al. [32], C3G has been found to stimulate the expression of endothelial nitric oxide synthase (eNOS) and enhance the production of nitric oxide (NO) in bovine vascular endothelial cells. This is believed to occur through regulating eNOS and Akt phosphorylation and the subsequent increase in cyclic guanosine monophosphate (cGMP) production [33]. Because of the significant capability of C3G to eliminate reactive oxygen species (ROS), Ziberna et al. [29] suggest that the indirect mechanisms associated with C3G ROS scavenging ability may be linked to the acute activation of eNOS. Fushimi et al. [34] state that a single 1 mg/kg dose of C3G orally increased rat cremaster arteriole blood flow and induced Akt phosphorylation in the aorta. According to the authors, C3G increases the fluid shear stress, which activates the vascular endothelial cell. This triggers the phosphorylation of Akt/eNOS, leading to the formation of NO. The proposed mechanisms of action concerning the vascular impacts of C3G highly emphasize the importance of the endothelium and the NO system for the vasorelaxant effect. This also explains why the impact is imperceptible when L-NAME is present in RIAR (Figure 9) or in MAPB (Figure 11) and the endothelium’s absence in RIAR (Figure 8).

The vasorelaxant effect of ME and C3G observed in RIAR was not affected by the administration of atropine and indomethacin, recognized as muscarinic receptor antagonists and prostanoid production inhibitors, respectively (Figure 9). The results show that the relaxation caused by ME and C3G in RIAR was not associated with muscarinic receptors or prostaglandin actions. It should be noted that the vasorelaxation induced by ACh is primarily a result of the stimulation of muscarinic receptors in endothelial cells. This leads to the activation of endothelial NO synthase and the subsequent enhancement of NO production [35]. Both ACh and C3G exhibit a vasorelaxant effect by activating the NO vascular system; however, in our investigation, their triggering mechanisms differ. According to our findings, the vasorelaxant effect of ME and C3G in RIAR is not mediated by cholinergic receptors or by the production of prostaglandins. Anthocyanins, like C3G, inhibit prostaglandin production by suppressing COX1/COX2 or enhancing COX2 expression. [36,37]. Based on this evidence, C3G might possess vasoconstrictor mechanisms, which could be induced by decreased prostaglandin production. Indomethacin, a COX inhibitor, would expose these mechanisms. However, in our experiments with RIAR, the vasorelaxant mechanisms overcome potentially vasoconstrictive mechanisms. In contrast, in anesthetized rats, the hypotensive effect and bradycardia caused by ME or C3G decreased with the pretreatment of indomethacin (Figure 7). Indomethacin induces an attenuation of the cardio-depressor effect produced by ME and C3G. This may be explained by the exposure of vascular smooth muscle cells to mechanical stretching forces. Administration of a single oral dose of C3G results in immediate hypotensive effects in Wistar rats. According to Fushimi et al. [34], this effect can be attributed to the vasodilation of skeletal muscle caused by C3G, which increases fluid shear stress, which activates vascular endothelial cells, and is activated through Akt/eNOS with the consequent increase in the production of NO. Alshihabi et al. [38] show that shear stress increases PGE2 and PGI2 release in vascular smooth muscle cells and that PGI2 is maximal right after the onset of shear and remains elevated for the first three hours. Moreover, the PGI2 release is more sensitive to shear stress at a level of 0.5 dynes/cm^2^. Our findings show that pre-treatment with indomethacin in rats leads to a decrease in the hypotension caused by ME or C3G. The mechanism underlying the indomethacin effect can be attributed to the suppression of prostaglandin production that is triggered by the rise in vascular fluid shear stress. In addition, Figure 7 shows that atropine pretreatment can attenuate the cardiovascular depressant effect. The involvement of the cholinergic system is complicated, and the determination of its mechanisms requires experimental studies.

The similarity between the effects of ACh and the observed cardiovascular depression of ME or C3G, as well as the vasorelaxant effect in RIAR, is clear. Even the influence of L-NAME on the reduction of MAP and HR in anesthetized rats, as shown in Figure 7, and on the vasodilatory effect of large (Figure 9) and small (Figure 11) blood vessels, produced by ME or C3G, is like that of ACh. The evidence shows that the relaxation of smooth muscle cells in blood vessels is induced by ACh through the activation of NO synthase and subsequent NO production [39,40]. The inhibition of NO synthase by L-NAME blocks this vasodilator mechanism [40]. However, studies conducted in vivo reveal that systemic arterial hypotension caused by ACh cannot be reduced by the inhibition of NO synthase through L-NAME [40,41]. ME or C3G vasorelaxation is inhibited by L-NAME in vitro (RIAR and MABR), while the hypotensive effect observed in vivo (MABR) is resistant. These facts can be partly explained by the reports of the impact of L-NAME on the vasorelaxant effect and the transient hypotension because of ACh [42,43]. However, we need to conduct new experiments to completely understand the mechanisms behind the hypotensive effects of ME and C3G.

## 4. Materials and Methods

### 4.1. Chemicals and Solutions

Methanol, chloroform, ethyl acetate, hydrochloric acid (36.5–38%), and amyl alcohol ACS reagents were purchased from JT BakerTM (Phillipsburg, NJ, USA). HPLC methanol and acetonitrile were obtained from LiChrosolv^®^ (Merck KGaA, Darmstadt, Germany) and JT BakerTM (Phillipsburg, NJ, USA), respectively. ACS reagents, including formic acid, kuromanin chloride (cyanidin 3-*O*-glucoside), (±)-Norepinephrine (+)-bitartrate salt, acetylcholine hydrochloride, L-NAME hydrochloride, indomethacin, atropine sulfate, prazosin, chloralose, and urethane, were purchased from Sigma-Aldrich (St. Louis, MO, USA). The water was purified by a Milli-Q^®^ system from Millipore (Bedford, MA, USA). We filtered the solvents for membrane filter Nylon, pore size 0.45 µm GVS (Apodaca, NL, México), and silica gel 60 M MACHEREY-NAGEL GmbH & Co. KG (Düren, Germany). The composition of the adapted Krebs solution was as follows (mM): NaCl, 130; KCl, 4.7; MgSO_4_, 1.2; KH_2_PO_4_, 0.16; CaCl_2_, 1.6; NaHCO_3_, 14.9; dextrose, 5.6; ascorbic acid, 0.6; it bubbled with a mixture of 95% O_2_ and 5% CO_2_ [44]. All salts were ACS reagents purchased from JT BakerTM (Phillipsburg, NJ, USA). Norepinephrine, acetylcholine, indomethacin, atropine, L-NAME, extracts, and fractions were diluted with NS solution for in vivo assays. All extracts and C3G were free of methanol or HCl. For in vitro trials, we diluted them in an adapted Krebs medium. The volume of injection was 1 mL/kg in whole rats. Indomethacin was mixed with sodium bicarbonate at 0.5% (*w*/*v*).

### 4.2. Plant Material

*Chiranthodendrun pentadactylon* Larreat is known in Spanish as “arbol de las manitas” [45] and in English as Devil’s Hand Tree, Hand Flower Tree, and Mexican Hand Plant [46]. We collected the flowers in Metepec, Mexico State, at the following coordinates: 19°15′48″N, 99°37′19.6″W. The Wildlife Conservation-Exploitation Management Unit, abbreviated as UMA, is designated as SEMARNAT-UMA-IN-325-MEX/21 (Supplementary Figure S1). The Official Mexican Standards NOM-059-SEMARNAT-2010 [47] have declared *Chiranthodendron pentadactylon* Larreat a threatened species. SEMARNAT allowed the Federal Delegation of the State of Mexico to acknowledge CHPE 1 as the ancestor of the UMA tree. We have deposited a specimen at the Faculty Herbarium of Sciences in UNAM, under the supervision of Dr. Jaime Jiménez Ramírez, who verified the plant material (reference No. 181307) (Supplementary Figure S2). The botanical designation of the *Chiranthodendron pentadactylon* Larreat is described on http://www.theplantlist.org (accessed on 15 May 2023) [48].

### 4.3. Extraction and Fractionation

The fresh flowers (248.8 g) were macerated at room temperature with methanol. This procedure was repeated 6 times (6 × 800 mL, 24 h each). The filtered extractions were combined, and the methanol was evaporated at a temperature not exceeding 40 °C, which resulted in a reddish-brown amorphous solid (22.3 g yield). This ME was later separated by liquid-liquid extraction with ethyl acetate and water (50:50). The organic (4.7 g yield) and inorganic (17.6 g yield) extracts were dried at room temperature and tested in the whole cardiovascular system of the rat. The aqueous fraction, which shows major evidence of cardiovascular activity, was partitioned in a chromatographic column of silica gel (60 M) at a 1:20 ratio. A solution of chloroform, methanol, and acetonitrile at a ratio of 2:1:1 was used as the mobile phase. The fractions were analyzed by thin-layer chromatography with a chloroform-methanol (2:1) mobile phase. Based on their chromatographic pattern, they were grouped to test their biological activity. Active fractions were subjected to chromatography until anthocyanin was identified as the active principle via the Rosenheim reaction [10].

### 4.4. Anthocyanin Extraction

To increase the stability of anthocyanin, 169 g of fresh flowers were macerated in acidified methanol (0.01% *v*/*v*) with HCl. This procedure was repeated 4 times (4 × 500 mL, 1 h each). The methanol was filtered and evaporated at 40 °C, resulting in 14.4 g of extract with a high content of anthocyanin. Part of this extract (20.9 mg) was dissolved in acidified water to 0.01% (*v*/*v*) with HCl and was loaded into the solid phase extraction (SPE) C18 (500 mg/6 mL cartridge (Finisterre^®^, Teknokroma, Barcelona, Spain), previously installed in the vacuum manifold (VisipredTM, Supelco Inc., Bellefonte, PA, USA). It was conditioned with two volumes of methanol, followed by three volumes of acidified water (0.01% *v*/*v*) with HCl. Unabsorbed substances were removed with 20 mL of acidified water (0.01% *v*/*v*) with HCl. Substances of low polarity were extracted with 20 mL of ethyl acetate [49]. The anthocyanin purified was recovered with acidified methanol (0.01% *v*/*v*) with HCl. This last solution was scanned by UV-Vis spectrometry to obtain the maximum absorbance.

### 4.5. Identification and Quantification of Anthocyanin

We use a Waters HPLC (Milford, MA, USA) system, including a 2487 dual UV/Vis absorbance detector, a 717 plus auto-sampler, and a 600 pump, to detect the purified anthocyanin. A volume of 10 µL of the anthocyanin sample was injected for separation in a column C18 (Agilent, ZORBAX, Santa Clara, CA, USA) column (4.6 × 150 mm^2^) with a particle size of 5 µm. It was eluted in mobile phases of water-formic acid (5% *v*/*v*) (phase A) and methanol-formic acid (5% *v/v*) (phase B). The gradient program was 15% B (0–2 min), 15–45% B (2–32 min), 45–15% B (32–33 min), and 15–0% B (40–63 min) with a flow rate of 0.8 mL/min [50]. The information was recorded at a wavelength of 520 nm. The identification of the purified anthocyanin was carried out by comparing its retention time value with that of the reference substance.

### 4.6. Sample and C3G-Standard Preparation

The purified anthocyanin and C3G standard were independently dissolved in 1 mL of acidified methanol (0.01% *v*/*v*) with HCl to create a solution with a 1 mg/mL concentration. The solutions were filtered using a syringe membrane filter, PVDF, pore size 0.45 µm (MS^®^, Shanghai, China), and placed inside an HPLC vial for analysis by HPLC. To establish the calibration curves, the C3G-standard solution was diluted to four concentrations: 0.025, 0.05, 0.075, and 0.1 μg/mL.

### 4.7. Animals

Male Wistar rats (250–350 g) were used for all experiments. The animals were maintained at room temperature (21–23 °C) on a 12 h light:12 h dark cycle and fed pellet food (5001 Rodent Laboratory Chow) ad libitum. Their care was in line with Mexican standards NOM-062-ZOO-1999, NOM-087-SEMARNAT-SSA1-2002 [51,52], and the Guide for the Care and Use of Laboratory Animals, revised in 2011 [53]. The procedures were approved by the Research Ethics Committee of the Faculty of Medicine at UNAM (Project No. 089-2009).

### 4.8. Pharmacological Experiments

#### 4.8.1. Effects of Increasing Doses of ME from the Flowers on the MAPHR

Dose-response curves were performed in rats anesthetized with a mixture of chloralose and urethane (50–800 mg/kg, i.p.). The experimental model implemented to record the changes in MAP and HR by several treatments was conducted according to Magos et al. [54]. To measure MAP and administer drugs, cannulas PE 50 (BD IntramedicTM, Sparks, MD, USA) were surgically inserted into the carotid artery and jugular vein. In the trachea, cannula PE 240 was inserted to facilitate spontaneous breathing. A pressure transducer model P231D (Gould-Statham Instruments, Oxnard, CA, USA) connected to the arterial cannula was used to record MAP. The signal from the transducer was electronically damped and registered by a Model 79 Grass polygraph (Grass Instrument, Quincy, MA, USA). HR was recorded through another channel from the polygraph with a Grass 7P4 tachograph triggered by the pulse waves from the unfiltered transducer signal, and the pressure change was transmitted to the software Lab-View^TM^ 21 SP1 (National Instruments, Austin, TX, USA) via an interface NI USB-6009 multifunction DAQ (National Instruments, Austin, TX, USA). The animal’s temperature was maintained at 38 °C using a heating table coupled to a regulation unit. All experiments used an electronic thermometer to regulate the temperature probe control of the heating table. After a stabilization period of about 15 min, successive injections of ME (1.7, 3.1, 5.6, 10, 17, 31, and 56 mg/kg, i.v.) or acetylcholine (ACh) (0.10, 0.17, 0.31, 0.56, 1.0, 1.7, 3.1, 5.6, and 10 µg/kg, i.v.) were administered. ACh was used to value the MAPHR reactivity. Each bolus injected was separated by a time interval to allow the recovery of the stability of MAP and HR. This procedure was repeated for each of the three treatments 6 times (six animals per treatment).

#### 4.8.2. Effects of One Dose of ME on the MAPHR

To determine the duration of cardiovascular effects, an additional group of six animals was administered a single dose of ME (56 mg/kg, i.v.). We chose this dose according to the dose–response curve, which is close to the maximum immediate hypotensive effect. To sum up, every group was integrated by six rats, and their MAP and HR were recorded 7 times over 60 min (before and every 10 min). We collected six values for each of those 7 recordings, from which we present an average.

#### 4.8.3. Selection of the Active Extract and Their Division

Once the mean arterial pressure (MAP) and heart rate (HR) were stabilized in the anesthetized rats, the cardiovascular activity of the ethyl acetate and aqueous extracts was examined. The administered dose of both extracts was 15 mg/kg, i.v. This dose is equivalent to DE50 from the ME curve obtained in anesthetized rats. The administered dose of ACh was 1.5 µg/kg. We selected the fraction that shows a higher cardiovascular effect for the study. We divided the selected extract into chromatographic columns and evaluated their effect on MAPHR. Six animals per treatment.

#### 4.8.4. Effects of Increasing Doses of C3G of the Flowers on the MAPHR

Response dose curves were conducted using the results obtained from six anesthetized rats for each treatment. Sequential administrations of C3G (at dosages of 1.0, 1.7, 3.1, 5.6, 10, 17, 3, and 56 mg/kg, i.v.) were administered. Each bolus injected was separated by a time interval sufficient to allow the recovery of the stability of MAP and HR. This procedure was repeated 6 times with each of the eight doses.

#### 4.8.5. Effects of a Single Dose of C3G of the Flowers on the MAPHR

The duration of cardiovascular effects of one dose of C3G from the flowers (56 mg/kg, i.v.) was determined in a group of six rats. In this group, the MAP and HR were recorded 7 times over 60 min (before and every 10 min). We collect six values for each of those 7 recordings, from which we present an average.

#### 4.8.6. Influence of L-NAME, Atropine, and Indomethacin on the Effects of C3G of the Flowers in MAPH

To further analyze the mechanism responsible for the hypotensive response, groups of six rats were pre-treated with L-NAME (10 mg/kg), atropine (1 mg/kg), or indomethacin (10 mg/kg) before receiving escalating doses of ME or C3G (1.7, 3.1, 5.6, 10, 17, 31, and 56 mg/kg, i.v.). Each bolus injected was separated by a time interval to allow the recovery of the stability of MAP and HR. This procedure was repeated for each of the three pretreatments 6 times.

#### 4.8.7. Effects of Increasing Doses of ME or C3G of the Flowers on RIAR

Rats were euthanized with sodium pentobarbital (120 mg/kg, i.p.). The experimental model implemented to record the changes in the vascular tone by several treatments was conducted according to Magos et al. [55]. The upper thoracic aorta was removed and cut into 0.5cm long segments. These pieces were hung into a 4 mL organ chamber between two nickel/chromium wire hooks. The connection between the baths and a Grass Model 79 polygraph (Grass Instrument, Quincy, MA, USA) was established by attaching one hook fastened to the bottom of the baths to a Grass FT03 force transducer. The software Lab-View^TM^ 21 SP1 (National Instruments, Austin, TX, USA) via an interface NI USB-6009 multifunction DAQ (National Instruments, Austin, TX, USA) was used to transmit the tension change. In all experiments, the baths contained adapted Krebs solution (pH 7.4), with the following composition of the adapted Krebs solution (mM): NaCl, 130; KCl, 4.7; MgSO_4_, 1.2; KH_2_PO_4_, 0.16; CaCl_2_, 1.6; NaHCO_3_, 14.9; dextrose, 5.6; and ascorbic acid, 0.6 [44]. The solution was bubbled with 95% O_2_ and 5% CO_2_, and the temperature was maintained at 37 °C. The preparations were subjected to 1g of resting tension, which was maintained constant throughout the experiments. A series of 5 stimulations with NE (0.1 µM) were performed during the 60 min stabilization period. The integrity of the vascular endothelium was evaluated by confirming that contracted rings experienced 80% relaxation upon administration of ACh (1 µM). The endothelium was removed mechanically by rubbing the lumen with steel, and its absence was confirmed when the relaxation by ACh was less than 20%. After achieving a plateau of contraction with NE (0.1 µM), cumulative concentration-response curves were generated using the ME (0.0031, 0.01, 0.031, 0.1, 0.31, 1, and 3.1 mg/mL), the C3G (0.001, 0.0031, 0.01, 0.031, 0.1, 0.31, and 1 mg/mL), or ACh (0.001, 0.0031, 0.01, 0.031, 0.1, 0.31, and 1 µg/mL) in RIAR, with or without endothelium. All experimental groups comprised eight aortic rings.

#### 4.8.8. Influence of L-NAME, Atropine, and Indomethacin on the Effects of ME or C3G of the Flowers in RIAR

RIAR with endothelium was incubated with L-NAME (100 µM), atropine (10 µM), or indomethacin (10 µM) for 20 min before the NE administration (0.1 µM). After the pretreatment, cumulative additions of increasing concentrations of ME (0.001, 0.056, 0.031, 0.17, 1.0, and 5.6 mg/mL) or C3G (0.001, 0.0031, 0.01, 0.031, 0.1, 0.31, and 1 mg/mL) were made. An additional positive control group received ACh. The responses were expressed as relaxed percents on the NE (0.1 µM) contraction in all cases. The experimental groups were integrated with eight rings.

#### 4.8.9. Effects of the Dose of ME or C3G of the Flowers on the MABR

In all rats, heparin was administered (1000 IU/kg, i.p.) before the euthanasia (sodium pentobarbital 120 mg/kg, i.p.). The MABR was prepared as described by Longhurst et al. [56]. A cannula PE 60 (BD Intra-medic, Sparks, MD, USA) was placed at the origin of the superior mesentery artery for perfusion of the adapted Krebs solution. The esophagus, intestinal tract, and superior mesenteric vein were sectioned, and the mesentery was separated to be extracted and carefully placed in gauze maintained at a temperature of 37 °C. A peristaltic pump drive model 5101 (Heidolph Instruments GmbH & Co. KG, Schwabach, Germany) was employed in the experiment featuring peristaltic action to ensure the continuous perfusion of the preparation with an adapted Krebs solution [44]. This Krebs solution was aerated with carbogen (95% O_2_ and 5% CO_2_) and maintained at 37 °C with a heated recirculation model 1104 (VWR^®^ International, LLC., Visalia, CA, USA) and at pH 7.4, with carbogen bubbling. The MABR was perfused at a rate of 2 mL/min to obtain an initial basal pressure of 15–25 mm Hg. To measure perfusion pressure variations, a P23XL pressure transducer (Gould-Statham Instruments, Oxnard, CA, USA) was connected to a Grass 79 polygraph (Grass Instrument, Quincy, MA, USA), and the pressure change was transmitted to the software Lab-View^TM^ 21 SP1 (National Instruments, Austin, TX, USA) via an interface NI USB-6009 multifunction DAQ (National Instruments, Austin, TX, USA). After 30 min of stabilization, increasing concentrations of NA (0.1, 0.31, 1.0, 3.1, 10, 31, and 100 µM) were administered before and after adding a concentration of ME (1 mg/mL) or C3G (0.1 mg/mL) to the perfusion solution. The effect of each concentration was measured in the steady state 5 min after the addition of each dose. Six experiments were performed for each product. Concentration-response curves for NA were generated based on the outcomes, both before and after the addition of ME or C3G to the adapted Krebs solution. Prazosin was used to evaluate the reactivity of the experimental model. L-NAME pretreatment (60 µM) on the effects of C3G on MABR was also studied.

### 4.9. Statistical Analysis

The experimental data are expressed as the mean ± standard error of the mean (SEM). MAP and HR are depicted in mm Hg and beats/min. Vascular relaxation is reported as a NE contraction inhibition percentage and perfusion pressure in mm Hg. The MAP, HR, relaxation, and perfusion pressure values were assessed and compared to their respective control groups and treatments. The statistical tests used were the Student’s *t*-test for paired and independent samples, as well as a one-way ANOVA Dunnett’s test for multiple comparisons. The effective dose 50 (DE_50_) and the inhibitory concentration 50 (IC_50_) were calculated through regression analysis on concentration–dose–response curves using GraphPad Prism 8.4.3 software (GraphPad Software Inc., San Diego, CA, USA). A probability level of less than 0.05 was considered significant in all cases.

## 5. Conclusions

The ME extract of *Chiranthodendron pentadactylon* flowers, along with its C3G component, induces cardiovascular depression in vivo and promotes vasorelaxation in vitro. We attribute the vasorelaxant effect to the generation of NO, without requiring cholinergic receptor activation or prostaglandin production. In contrast, these signaling pathways lead to cardiovascular depression in anesthetized rats. Our results contribute to the progress of knowledge regarding the ethnomedicinal use of flower-derived products for blood pressure treatment. They possess significant social implications as they show that the administration of C3G could cause cardiovascular effects similar to those of a complete extract derived from *Chiranthodendron pentadactylon* flowers. This provides an opportunity for the community consuming the flower to prioritize the preservation of a threatened species. The rational use of the plant should be to promote and research the pharmacology of C3G alone or in combination with other natural products. Further investigation is needed to conduct bio-prospecting studies and establish the importance of the traditional uses of this plant. It is necessary to provide evidence concerning the safety profile of the plant to evaluate the potential hazards linked to the consumption of the flower. The magnitude of these hazards can either enhance or diminish the medicinal value of this species. The combination of extensive research and evaluation of various extracts derived from *Chiranthodendron pentadactylon* flowers in multiple cardiovascular system models, including models of arterial hypertension in conscious and anesthetized rats, will ascertain the genuine medicinal and toxicological significance of the plant.

## Figures and Tables

**Table 1:** Baseline values of MAP and HR were recorded before the administration of Ach, ME and C3G in anesthetized rats.

Treatment	MAP (mm Hg)	HR (Beats/min)
Control (ACh)	138.8 ± 11.7	318.7 ± 9.3
ME	152.4 ± 25.1	426.6 * ± 5.5
C3G	136.4 ± 2.9	331.8 ± 8.5

MAP: mean arterial pressure. HR: heart rate. Acetylcholine (ACh). Data shown represent means ± SD, *n* = 6, for each treatment. * *p* < 0.0001 for the control (ACh) vs. ME, Student’s t-test for two independent samples.

**Table 2:** Baseline values of MAP and HR were recorded before the administration of NS, ME, and C3G in anesthetized rats.

Treatment	MAP (mm Hg)	HR (Beats/min)
Control (NS)	123.8 ± 29.2	344.8 ± 34.8
ME	129.4 ± 11.0	358.2 ± 25.5
C3G	148.8 ± 32.9	361.6 ± 14.1

MAP: mean arterial pressure. HR: heart rate. NS: normal saline. Data shown represent means ± SD, n = 6, for each treatment.

**Table 3:** Baseline values of MAP and HR were recorded before the administration of Ach, Ethyl acetate extract, and Aqueous extract in anesthetized rats.

Treatment	MAP (mm Hg)	HR (Beats/min)
NS	151.1 ± 22.0	456.6 ± 6.0
Control (ACh)	138.5 ± 15.6	450.2 ± 8.4
Ethyl acetate extract	141.4 ± 11.3	456.3 ± 6.9
Aqueous extract	142.5 ± 10.9	473.1 ± 24.2

MAP: mean arterial pressure. HR: heart rate. NS: normal saline. Acetylcholine (ACh). Data shown represent means ± SD, n = 6, for each treatment.

**Table 4:** Initial values of MAP and HR in the diverse pretreatment of anesthetized rats.

Pretreatment	MAP (mm Hg)	HR (Beats/min)
Control (C3G)	136.4 ± 2.9	331.8 ± 8.5
C3G + L-NAME	181.6 * ± 13.1	321.5 ± 7.4
C3G + Atropine	134.3 ± 6.3	310.1 ± 11.4
C3G + Indomethacin	145.8 ± 16.8	323.9 ± 6.6

MAP: mean arterial pressure. HR: heart rate. C3G: Cyanidin 3-O glucoside. Data shown represent means ± SD, *n* = 6, for each treatment. * *p* < 0.0001 for the control (ACh) vs. C3G + L-NAME, Student’s t-test for two independent samples.

## Data Availability

Data are contained within the article and supplementary materials.

## Supplementary Materials

The following supporting information can be downloaded at: https://www.mdpi.com/article/10.3390/molecules28237698/s1.

## Abbreviations

Acetylcholine (ACh); Cyanidin 3-*O*-glucoside (C3G); Cyclooxygenase (COX); Effective doses 50 (DE50); Endothelial nitric oxide synthase (eNOS); Cyclic guanosine monophosphate (cGMP); Heart rate (HR); High-performance liquid chromatography (HPLC); Inhibitory concentration 50 (IC50); Intravenous (i.v.); Mean arterial pressure (MAP); Mean arterial pressure-heart rate (MAPHR); Mesenteric arterial bed (MABR); Methanolic extract (ME); Nitric oxide (NO); Norepinephrine (NE); Normal saline (NS); Nω-Nitro-L-arginine methyl ester (L-NAME); Rat isolated aortic ring (RIAR); Prostaglandin E2 (PGE2); Prostaglandin I2 (PGI2); Protein kinase B (Akt); Reactive oxygen species (ROS); Standard error of the mean (SEM); Ultraviolet-visible (UV-Vis); Fresh weight (FW).
