# Supplementary material for: The Hypotensive and Vasodilatory Effects Observed in Rats Exposed to Chiranthodendron pentadactylon Larreat Flowers Can Be Attributed to Cyanidin 3-O-Glucoside
Source: Molecules. 2023 Nov 22;28(23):7698. doi: 10.3390/molecules28237698 (PMC10707424; doi:10.3390/molecules28237698)
Supplement: Supplementary file 1 [file molecules-28-07698-s001.zip › molecules-2708016-supplementary.pdf]

Supplementary Data

The Hypotensive and Vasodilatory Effects Observed in Rats Exposed to *Chiranthodendron pentadactylon* Larreat Flowers Can Be Attributed to Cyanidin 3-*O*-Glucoside

Juan Luis Escobar-Ramírez, Jacinto Santiago-Mejía, Maribel Soto-Núñez, Oscar Salvador Barrera-Vázquez , Roberto Vargas-Querea and Gil Alfonso Magos-Guerrero *

Department of Pharmacology, Faculty of Medicine, University National Autonomous of Mexico, Mexico City 04510, Mexico; re-harakhty@hotmail.com (J.L.E.-R.); samj@unam.mx (J.S.-M.); farma_estrogenos@yahoo.com (M.S.-N.); osbarrera6@gmail.com (O.S.B.-V.); gcomrv@gmail.com (R.V.-Q.)

* Correspondence: gamagos@unam.mx; Tel.: +52-55-5623-2165

This file includes:

1. Supplementary Tables S1.1–S8.1. Detailed experiment data.

2. Supplementary Figure S1. Register management unit for the conservation of wildlife known by its acronym in Spanish UMA.

3. Supplementary Figure S2. Herbarium specimen with the number 181307 collected in: Paseo Sta. Ana #142, San Carlos, Metepec, Edo de México.

2.2. Effects of increasing doses of ACh, ME, or C3G on the MAPHF

Table S1.1. Effects of increasing doses of ACh, ME or C3G on the MAP (mm Hg) of anesthetized rat.

Treatment	Dose (μg/Kg)								
	0.10	0.17	0.31	0.56	1.0	1.7	3.1	5.6	10.0
Control (ACh) (μg/kg)	0	-10.02 ± 2.40	-20.66 ± 1.60	-27.41 ± 2.15	-33.75 ± 4.26	-36.37 ± 4.64	-40.57 ± 3.79	-45.13 ± 3.27	-53.23 ± 5.02
	Dose (mg/Kg)								
	1	1.7	3.1	5.6	10.0	17.0	31.0	56.0	100.0
ME (mg/Kg)	-	-	-2.04 ± 1.85*	-7.40 ± 5.14	-11.00 ± 2.04*	-29.40 ± 3.94	-60.20 ± 6.14	-60.25 ± 6.73	-
C3G (mg/Kg)	0	-10.25 ± 2.52	-19.70 ± 4.64	-26.83 ± 5.37	-37.52 ± 5.13	-40.68 ± 4.75	-48.05 ± 3.85	-49.93 ± 3.36	-

ACh: Acetylcholine; ME: Methanolic extract; C3G: Cyanidin 3-*O*-glucoside; MAP: Mean Arterial Pressure. Data showed represent means ± SEM; n = 6.

* p = 0.001 for Control (ACh) vs ME; Dose 3.1 (mg/Kg).

* p = 0.017 for Control (ACh) vs ME; Dose 10 (mg/Kg).

Treatment / Dunnett (bilateral) / Analysis of the differences between the treatment Control (Ach) category vs ME and C3G categories with a 95% confidence interval.

Table S1.2. Effects of increasing doses of ACh, ME or C3G on the HR (Beats/min) of anesthetized rat.

Treatment	Dose (μg/Kg)								
	0.10	0.17	0.31	0.56	1.0	1.7	3.1	5.6	10.0
Control (ACh) (μg/kg)	0	0	-1.75 ± 1.10	-2.38 ± 1.48	1.43 ± 2.24	-3.64 ± 1.03	-7.52 ± 2.39	-36.73 ± 12.63	-72.95 ± 10.24
	Dose (mg/Kg)								
	1	1.7	3.1	5.6	10.0	17.0	31.0	56.0	100.0
ME (mg/Kg)	-	-	-6.0 ± 3.95	-4.0 ± 0.88	-12.20 ± 7.43	-23.20 ± 13.52	-63.75 ± 11.13	-61.50 ± 13.26	-
C3G (mg/Kg)	0	-1.85 ± 2.45	-5.65 ± 2.06	-7.88 ± 3.30	-29.95 ± 9.46	-45.55 ± 10.54	-43.69 ± 12.20	-113.55 ± 13.22[#]	-

ACh: Acetylcholine; ME: Methanolic extract; C3G: Cyanidin 3-*O*-glucoside; HR: Heart rate. Data showed represent means ± SEM; n = 6.

[#] p = 0.041 for Control (Ach) vs C3G; Dose 56.0 (mg/Kg).

Treatment / Dunnett (bilateral) / Analysis of the differences between the treatment Control (Ach) category vs ME and C3G categories with a 95% confidence interval.

2.3. Effects of a single dose of ME or C3G on the MAPHF

Table S2.1. Effects of a single dose of NS, ME and C3G on the MAP (mm Hg) of anesthetized rat.

Treatment	Time (min)						
	0	10	20	30	40	50	60
Control (NS) (mL/Kg)	0	-6.03 ± 0.95	-0.5 ± 5.50	-7.50 ± 1.50	-8.04 ± 1.00	-8.50 ± 2.50	-1.52 ± 6.50
ME (mg/Kg)	0	-32.27 ± 3.10#	-27.06 ± 5.00#	-24.59 ± 6.80#	-26.88 ± 7.50#	-26.80 ± 7.60	-32.07 ± 8.20
C3G (mg/Kg)	0	-50.21 ± 3.50*	-35.43 ± 3.00*	-15.36 ± 6.00	-17.48 ± 5.75	-19.81 ± 3.75	-15.64 ± 4.25

NS: Normal saline; ME: Methanolic extract; C3G: Cyanidin 3-*O*-glucoside; MAP: Mean Arterial Pressure. Data showed represent means ± SEM; $n = 6$.

# p = 0.032 for Control (NS) vs ME; Time 10.

* p = 0.004 for Control (NS) vs C3G; Time 10.

# p = 0.027 for Control (NS) vs ME; Time 20.

* p = 0.016 for Control (NS) vs C3G; Time 20.

# p = 0.045 for Control (NS) vs ME; Time 30.

# p = 0.048 for Control (NS) vs ME; Time 40.

Treatment / Dunnett (bilateral) / Analysis of the differences between the treatment Control (NS) category vs ME and C3G categories with a 95% confidence interval.

Table S2.2. Effects of a single dose of NS, ME and C3G on the HR (Beats/min) of anesthetized rat.

Treatment	Time (min)						
	0	10	20	30	40	50	60
Control (NS) (mL/kg	0	-4.74 ± 4.40	-4.74 ± 5.24	-2.49 ± 5.67	-6.65 ± 2.20	-4.44 ± 3.30	-4.80 ± 4.27
ME (mg/Kg)	0	-5.83 ± 7.75	-4.83 ± 3.49	-24.83 ± 4.29#	-13.17 ± 4.49	1.67 ± 3.06	2.50 ± 3.06
C3G (mg/Kg)	0	-3.06 ± 3.31	-6.60 ± 4.49	-11.70 ± 7.51	-6.47 ± 8.76	-11.42 ± 3.85	-11.64 ± 4.49

NS: Normal saline; ME: Methanolic extract; C3G: Cyanidin 3-*O*-glucoside; HR: Heart rate. Data showed represent means ± SEM; $n = 6$.

# p = 0.040 for Control (NS) vs ME; Time 30.

Treatment / Dunnett (bilateral) / Analysis of the differences between the treatment Control (NS) category vs ME and C3G categories with a 95% confidence interval.

2.4. Selection of the aqueous fraction

Table S3.1. Effects of a single dose of NS, ACh, Ethyl acetate fraction and Water fraction on the MAP (mm Hg), and HR (Beats/min) of anesthetized rat.

Treatment	MAP (mm Hg)	HR (Beats/min)
NS	2.90 ± 2.00#	3.30 ± 2.23
Control (ACh)	-36.3 ± 4.64	-4.00 ± 1.03
Ethyl acetate extract	-2.44 ± 1.26*	-2.67 ± 0.97
Aqueous extract	-25.6 ± 4.83	-6.43 ± 6.73

NS: Normal saline; ACh: Acetylcholine; MAP: Mean Arterial Pressure; HR: Heart rate. Data showed represent means ± SEM; *n* = 6.

# $p < 0.0001$ for Control (ACh) vs NS.

* $p < 0.0001$ for Control (ACh) vs Ethyl acetate fraction.

Treatment / Dunnett (bilateral) / Analysis of the differences between the treatment Control (Ach) category vs NS, Ethyl acetate fraction, and Water fraction categories with a 95% confidence interval.

2.5. Influence of L-NAME, atropine, and indomethacin on the effects of C3G in the MAPHF

Table S4.1. Effects of L-NAME, Atropine, and Indomethacin on the effects of C3G on the MAP (mm Hg) of anesthetized rat.

Treatment	Dose (mg/Kg)						
	1.7	3.1	5.6	10.0	17.0	31.0	56.0
Control (C3G)	-10.25 ± 2.52	-19.70 ± 4.64	-26.83 ± 5.37	-37.52 ± 5.13	-40.68 ± 4.75	-48.05 ± 3.85	-49.93 ± 3.36
C3G+ L-NAME (10 mg/Kg)	0	-4.01 ± 1.78∞	-21.56 ± 5.84	-30.26 ± 6.29	-46.04 ± 7.42	-58.98 ± 6.71	-63.51 ± 5.23
C3G+ Atropine (1 mg/Kg)	0	2.65 ± 3.25*	-2.21 ± 4.44*	-17.55 ± 2.78	-20.36 ± 1.56	-26.77 ± 4.27	-32.25 ± 5.52*
C3G+ Indomethacin (10 mg/Kg)	0	-6.98 ± 1.88#	-8.04 ± 1.85	-12.66 ± 4.12#	-19.88 ± 6.77	-33.95 ± 13.41	-20.01 ± 4.61#

L-NAME; Nω-Nitro-L-arginine methyl ester; C3G: Cyanidin 3-*O*-glucoside; MAP: Mean Arterial Pressure. Data showed represent means ± SEM, *n* = 6.

∞ p = 0.002 Control (C3G) vs C3G+L-NAME D 3.1 (mg/Kg).

* p = 0.0002 for Control (C3G) vs C3G+Atropine; Concentration 3.1 (mg/Kg).

\# p = 0.012 for Control (C3G) vs C3G+Indomethacin; Concentration 3.1 (mg/Kg).

* p = 0.004 for Control (C3G) vs C3G+Atropine; Concentration 5.6 (mg/Kg).

\# p = 0.013 for Control (C3G) vs C3G+Indomethacin; Concentration 10.0 (mg/Kg).

* p = 0.030 for Control (C3G) vs C3G+Atropine; Concentration 56.0 (mg/Kg).

\# p = 0.001 for Control (C3G) vs C3G+Indomethacin; Concentration 56.0 (mg/Kg).

Treatment / Dunnett (bilateral) / Analysis of the differences between the treatment Control (C3G) category vs C3G + L-NAME, C3G + Atropine and C3G + Indomethacin categories with a 95% confidence interval.

Table S4.2. Effects of L-NAME, Atropine, and Indomethacin on the effects of C3G on the HR (Beats/min) of anesthetized rat.

Treatment	Dose (mg/Kg)						
	1.7	3.1	5.6	10.0	17.0	31.0	56.0
Control (C3G)	-1.85 ± 2.45	-5.65 ± 2.06	-7.88 ± 3.30	-29.95 ± 9.46	-45.55 ± 10.54	-43.69 ± 12.20	-113.55 ± 13.22
C3G+ L-NAME (10 mg/Kg)	0	-2.66 ± 1.75	-10.44 ± 5.16	-19.5 ± 9.79	-50.76 ± 16.47	-79.25 ± 19.27	-95.75 ± 17.31
C3G+ Atropine (1 mg/Kg)	-2.1 ± 2.1	-11.71 ± 3.41	-10.62 ± 3.08	-15.53 ± 4.67	-18.35 ± 6.43	-12.40 ± 8.89	-24.19 ± 7.85*
C3G+ Indomethacin (10 mg/Kg)	-1.2 ± 1.1	-10.78 ± 3.43	-6.16 ± 14.13	-15.07 ± 4.05	-14.53 ± 3.09	-17.56 ± 7.59	-21.08 ± 7.60#

L-NAME; Nω-Nitro-L-arginine methyl ester; C3G: Cyanidin 3-*O*-glucoside; HR: Heart rate. Data showed represent means ± SEM, *n* = 6.

* p < 0.0001 for Control (C3G) vs C3G+Atropine; Concentration 56.0 (mg/Kg).

\# p < 0.0001 for Control (C3G) vs C3G+Indomethacin; Concentration 56.0 (mg/Kg).

Treatment / Dunnett (bilateral) / Analysis of the differences between the treatment Control (C3G) category vs C3G + L-NAME, C3G + Atropine, and C3G + Indomethacin categories with a 95% confidence interval.

2.6. Effects of increasing doses of ME or C3G on RIAR

Table S5.1. Effect of ACh and ME at different percentages of endothelium in the rat-isolated aortics rings.

Treatment	Concentration (μg/mL)						
	0.0031	0.01	0.031	0.1	0.31	1.0	3.1
Control (ACh)	-3.59 ± 2.34	-8.05 ± 2.21	-37.61 ± 2.27	-58.09 ± 2.59	-80.15 ± 1.98	-84.66 ± 1.39	-87.43 ± 1.69
	Concentration (mg/mL)						
	0.0031	0.01	0.031	0.1	0.31	1.0	3.1
ME (E<20%)	0	0*	-2±1.42*	-4±1.22*	-7±1.52*	-8±1.39*	-13±1.21*
ME (E>80%)	0	-3.75±1.85	-14±2.57#	-40±2.60#	-64±2.16#	-83±2.22	-90±0.84

ACh: Acetylcholine; ME: Methanolic extract. Data showed represent means ± SEM, *n* = 8.

* $p < 0.0001$ for Control (ACh) vs ME (E<20%); Concentration 0.01 (mg/Kg).

* $p < 0.0001$ for Control (ACh) vs ME (E<20%); Concentration 0.031 (mg/Kg).

# $p = 0.012$ for Control (ACh) vs ME (E>80%); Concentration 0.031 (mg/Kg).

* $p < 0.0001$ for Control (ACh) vs ME (E<20%); Concentration 0.1 (mg/Kg).

# $p = 0.019$ for Control (ACh) vs ME (E>80%); Concentration 0.1 (mg/Kg).

* $p < 0.0001$ for Control (ACh) vs ME (E<20%); Concentration 0.31 (mg/Kg).

# $p = 0.016$ for Control (ACh) vs ME (E>80%); Concentration 0.31 (mg/Kg).

* $p < 0.0001$ for Control (ACh) vs ME (E<20%); Concentration 1.0 (mg/Kg).

* $p < 0.0001$ for Control (ACh) vs ME (E<20%); Concentration 3.1 (mg/Kg).

Treatment / Dunnett (bilateral) / Analysis of the differences between the treatment Control (ACh) category vs ME Endothelium<20 % and ME Endothelium>80 % categories with a 95% confidence interval.

Table S5.2. Effect of ACh and C3G at different percentages of endothelium in the rat-isolated aortics rings.

Treatment	Concentration (μg/mL)						
	0.001	0.0031	0.01	0.031	0.1	0.31	1.0
Control (ACh)	-0.74 ± 1.61	-9.3 ± 2.92	-29.12 ± 3.42	-57.21 ± 3.43	-76.01 ± 2.76	-85.54 ± 2.84	-91.77 ± 2.43
	Concentration (mg/mL)						
	0.001	0.0031	0.01	0.031	0.1	0.31	1.0
C3G (E<20%)	-1.95 ± 1.84	-2.90 ± 4.12	-4.13 ± 4.01*	-2.68 ± 1.92*	-3.50 ± 3.35*	-9.16 ± 3.73*	-10.66 ± 5.24*
C3G (E>80%)	0.62 ± 1.37	-13.27 ± 7.45	-30.83 ± 5.95	-55.28 ± 6.14	-82.05 ± 4.15	-90.46 ± 4.06	-91.77 ± 5.47

ACh: Acetylcholine; C3G: Cyanidin 3-*O*-glucoside. Data showed represent means ± SEM, *n* = 8.

* $p = 0.006$ for Control (C3G) vs C3G (E<20%); Concentration 0.01 (mg/Kg).

* $p < 0.0001$ for Control (C3G) vs C3G (E<20%); Concentration 0.031 (mg/Kg).

* $p < 0.0001$ for Control (C3G) vs C3G (E<20%); Concentration 0.1 (mg/Kg).

* $p < 0.0001$ for Control (C3G) vs C3G (E<20%); Concentration 0.31 (mg/Kg).

* $p < 0.0001$ for Control (C3G) vs C3G (E<20%); Concentration 1.0 (mg/Kg).

Treatment / Dunnett (bilateral) / Analysis of the differences between the treatment Control (ACh) category vs C3G Endothelium<20% and C3G Endothelium>80% categories with a 95% confidence interval.

2.7 Influence of L-NAME, atropine, and indomethacin on the effects of ME or C3G of the flowers

Table S6.1. Effects of L-NAME, atropine and indomethacin on ME vasorelaxation in the rat-isolated aortics rings.

Treatment	Concentration (μg/mL)					
	0.001	0.0056	0.031	0.17	1.0	5.6
Control (ACh)	-1.56 ± 2.10	-4.03 ± 1.97	-25.21 ± 5.67	-50.94 ± 4.59	-72.62 ± 4.85	-82.95 ± 4.22
	Concentration (mg/mL)					
	0.001	0.0056	0.031	0.17	1.0	5.6
ME	-3.75 ± 0.86	-13.97 ± 1.66	-40.11 ± 1.56	-63.76 ± 1.22	-83.32 ± 1.21	-90.01 ± 0.80
ME+L-NAME (100 μM)	0	-2.04 ± 1.20	-3.92 ± 2.17*	-6.88 ± 3.01*	-9.11 ± 1.45*	-8.96 ± 3.11*
ME+Indomethacin (10 μM)	0	-16.11 ± 4.10	-44.11 ± 3.67	-59.92 ± 2.97	-75.88 ± 2.56	-87.87 ± 3.87
ME+Atropine (10 μM)	0	-14.13 ± 2.54	-40.13 ± 4.80	-63.67 ± 2.72	-72.69 ± 4.02	-85.75 ± 4.33

ACh: Acetylcholine; L-NAME: Nω-Nitro-L-arginine methyl ester; ME: Methanolic extract. Data showed represent means ± SEM, *n* = 8.

* p = 0.008 for Control (ACh) vs ME+L-NAME; Concentration 0.031 (mg/Kg).
* p < 0.0001 for Control (ACh) vs ME+L-NAME; Concentration 0.17 (mg/Kg).
* p < 0.0001 for Control (ACh) vs ME+L-NAME; Concentration 1.0 (mg/Kg).
* p < 0.0001 for Control (ACh) vs ME+L-NAME; Concentration 5.6 (mg/Kg).

Treatment / Dunnett (bilateral) / Analysis of the differences between the treatment Control (ACh) category vs ME + L-NAME, ME + Indomethacin and ME + Atropine with a 95% confidence interval.

Table S6.2. Effects of L-NAME, atropine and indomethacin on C3G vasorelaxation in the rat-isolated aortics rings.

Treatment	Concentration (μg/mL)						
	0.001	0.0031	0.01	0.031	0.1	0.31	1.0
Control (ACh)	-3.59 ± 1.95	-8.05 ± 2.70	-37.61 ± 2.52	-58.09 ± 3.31	-80.15 ± 2.94	-84.66 ± 1.91	-87.43 ± 1.76
	Concentration (mg/mL)						
	0.001	0.0031	0.01	0.031	0.1	0.31	1.0
C3G	0.62 ± 1.37	-13.27 ± 7.45	-30.83 ± 5.95	-55.28 ± 6.14	-82.05 ± 4.15	-90.46 ± 4.06	-91.77 ± 5.47
C3G+L-NAME (100 μM)	0.94 ± 0.79	-0.69 ± 1.92	-4.99 ± 3.53*	-13.02 ± 5.28*	-10.31 ± 5.70*	-9.22 ± 5.98*	-2.28 ± 4.50*
C3G+Indomethacin (10 μM)	-1.03 ± 1.18	-7.91 ± 2.30	-41.62 ± 4.84	-72.59 ± 2.02	-80.19 ± 1.39	-83.84 ± 1.05	-86.97 ± 1.29
C3G+Atropine (10 μM)	-1.80 ± 1.96	-7.22 ± 1.82	-59.92 ± 7.31	-75.86 ± 6.76	-84.65 ± 5.29	-84.98 ± 5.73	-88.85 ± 6.83

ACh: Acetylcholine; L-NAME: Nω-Nitro-L-arginine methyl ester; C3G: Cyanidin 3-*O*-glucoside. Data showed represent means ± SEM, *n* =8.

* p = 0.001 for Control (ACh) vs C3G+L-NAME; Concentration 0.01 (mg/Kg).
* p < 0.0001 for Control (ACh) vs C3G+L-NAME; Concentration 0.031 (mg/Kg).
* p < 0.0001 for Control (ACh) vs C3G+L-NAME; Concentration 0.1 (mg/Kg).
* p < 0.0001 for Control (ACh) vs C3G+L-NAME; Concentration 0.31 (mg/Kg).
* p < 0.0001 for Control (ACh) vs C3G+L-NAME; Concentration 1.0 (mg/Kg).

Treatment / Dunnett (bilateral) / Analysis of the differences between the treatment Control (ACh) category vs C3G + L-NAME, C3G + Indomethacin and C3G + Atropine with a 95% confidence interval.

2.8. Effects of a single dose of ME or C3G of the flowers on the MABR

Table S7.1. Effects of a single dose of ME in the rat mesenteric arterial bed.

Treatment	Concentration (μM)						
	0.1	0.31	1.0	3.1	10.0	31.0	100.0
Control (NE)	3.60 ± 0.49	6.00 ± 0.50	16.60 ± 1.19	30.80 ± 1.37	57.80 ± 2.41	77.60 ± 3.16	94.40 ± 5.44
NE+ME (1 mg/mL)	2.40 ± 0.37	4.40 ± 0.37*	9.20 ± 0.45*	18.00 ± 1.38*	29.20 ± 1.59*	40.40 ± 1.14*	63.00 ± 2.74*
NE+Prazosin (1 nM)	0.00 ± 1.56	5.67 ± 2.75	8.17 ± 2.96	12.00 ± 2.68	17.67 ± 2.16	22.33 ± 2.40	29.50 ± 1.78

NE; Norepinephrine; ME: Methanolic extract. Data showed represent means ± SEM, *n* = 6.
* p = 0.035 for Control (NE) vs NE+ME; Concentration 0.31 (μM).
* p = 0.030 for Control (NE) vs NE+ME; Concentration 1.0 (μM).
* p = 0.007 for Control (NE) vs NE+ME; Concentration 3.1 (μM).
# p = 0.002 for Control (NE) vs NE+Prazosin; Concentration 3.1 (μM).
* p = 0.0007 for Control (NE) vs NE+ME; Concentration 10.0 (μM).
# p < 0.0001 for Control (NE) vs NE+Prazosin; Concentration 10.0 (μM).
* p = 0.0003 for Control (NE) vs NE+ME; Concentration 31.0 (μM).
# p < 0.0001 for Control (NE) vs NE+Prazosin; Concentration 31.0 (μM).
* p = 0.0042 for Control (NE) vs NE+ME; Concentration 100.0 (μM).
# p < 0.0001 for Control (NE) vs NE+Prazosin; Concentration 100.0 (μM).
Related two-sample t-test / Two-sided test with a 95% confidence interval for the difference between the means.

Table S7.2. Effects of a single dose of C3G in the rat mesenteric arterial bed.

Treatment	Concentration (μM)						
	0.1	0.31	1.0	3.1	10.0	31.0	100.0
Control (NE)	4.00 ± 0.80	8.50 ± 1.11	12.00 ± 1.00	21.33 ± 1.96	50.17 ± 2.65	92.67 ± 3.13	126.33 ± 5.25
NE+C3G (0.1 mg/mL)	3.67 ± 0.78	7.33 ± 0.90	13.33 ± 0.98	19.50 ± 1.59*	31.33 ± 3.02*	56.50 ± 3.10*	77.00 ± 3.35*
NE+Prazosin (1 nM)	3.67 ± 0.63	5.67 ± 1.12	8.17 ± 1.21	12.00 ± 1.10	17.67 ± 0.88	22.33 ± 0.98	29.50 ± 0.73

NE; Norepinephrine; C3G: Cyanidin 3-*O*-glucoside. Data showed represent means ± SEM, *n* = 6.
# p = 0.022 for Control (NE) vs C3G+Prazosin; Concentration 3.1 (μM).
* p = 0.0015 for Control (NE) vs NE+C3G; Concentration 10.0 (μM).
# p = 0.0027 for Control (NE) vs NE+Prazosin; Concentration 10.0 (μM).
* p = 0.032 for Control (NE) vs NE+C3G; Concentration 31.0 (μM).
# p < 0.0001 for Control (NE) vs NE+Prazosin; Concentration 31.0 (μM).
* p = 0.0047 for Control (NE) vs NE+C3G; Concentration 100.0 (μM).
# p < 0.0001 for Control (NE) vs NE+Prazosin; Concentration 100.0 (μM).
Related two-sample t-test / Two-sided test with a 95% confidence interval for the difference between the means.

2.9. Influence of L-NAME in the effects of C3G of the flowers on the MABR

Table S8.1. Effects of L-NAME on C3G vasorelaxation in the rat mesenteric arterial bed.

Treatment	Concentration (μM)						
	0.1	0.31	1.0	3.1	10.0	31.0	100.0
Control (NE)	4.00 ± 1.46	10.17 ± 3.22	15.33 ± 3.77	26.17 ± 5.96	52.83 ± 10.93	113.33±15.12	162.67±9.60
NE+C3G (0.1 mg/mL)	2.83 ± 0.79	8.17 ± 2.40	13.67 ± 3.13	24.17 ± 6.82	40.33±7.01	58.33±6.61*	69.17±4.70*
NE+C3G (0.1mg/mL) + L-NAME (60 μM)	6.33 ± 0.49	14.33 ± 0.96	22.00 ± 1.57	44.50 ± 3.85	88.50±8.81	128.17±7.11	163.33±3.34

Nω-Nitro-L-arginine methyl ester; NE; Norepinephrine; C3G: Cyanidin 3-O-glucoside. Data showed represent means ± SEM, n = 6.

* $p = 0.004$ for Control (NE) vs NE+C3G; Concentration 31.0 (μM).

* $p < 0.0001$ for Control (NE) vs NE+C3G; Concentration 100.0 (μM).

Related two-sample t-test / Two-sided test with a 95% confidence interval for the difference between the means.

MEDIO AMBIENTE

Delegación Federal de SEMARNAT
en el Estado de México
Subdelegación de Gestión para la
Protección Ambiental y Recursos Naturales

DFMARNAT/0706/2021
Toluca, México a 24 de febrero de 2021

REGISTRO PARA EL ESTABLECIMIENTO DE UNA UNIDAD DE MANEJO PARA LA CONSERVACIÓN DE LA VIDA SILVESTRE (UMA).

NOMBRE O RAZÓN SOCIAL: C. DAVID GUAJARDO RUZ

Con fundamento en los artículos 27 tercer párrafo de la Constitución Política de los Estados Unidos Mexicanos; 26 y 32 Bis Fracciones I, III, y XXXIX de la *Ley Orgánica de la Administración Pública Federal*; 1, 2 Fracción XXX, 38, 39 y 40 Fracción IX -inciso i- y XIX del Reglamento Interior de la Secretaría de Medio Ambiente y Recursos Naturales; 79, 80, 82, 83, 86, y 87 de la Ley General del Equilibrio Ecológico y la Protección al Ambiente; 9 Fracción XI, 27, 28, 39, 40, 41 y 42 de la Ley General de Vida Silvestre; 12, 30, 31, 32, 33, 34, 35, 36 y Noveno Transitorio, del Reglamento de la Ley General de Vida Silvestre; y en virtud de haber cumplido con los ordenamientos vigentes en la materia, esta Delegación Federal otorga con vigencia INDEFINIDA el REGISTRO a la siguiente UMA:

**NOMBRE DE LA UMA:**	**"FLOR MANITA"**
**UBICACIÓN:**	**PASEO SANTA ANA #142., SAN CARLOS, C.P. 52159, METEPEC, ESTADO DE MÉXICO.**
**SUPERFICIE AUTORIZADA:**	**0.22 HECTÁREAS**
**CLAVE DE REGISTRO:**	**SEMARNAT-UMA-IN-325-MEX/21**
**TENENCIA DE LA TIERRA:**	**PRIVADA**
**TIPO DE PROPIEDAD:**	**PROPIEDAD X RENTADA_ EN COMODATO_ P/PODER_ OTRO_**
**FINALIDAD DE LA UMA:**	**REPRODUCCIÓN, EDUCACIÓN AMBIENTAL, , INVESTIGACIÓN, EXHIBICIÓN Y APROVECHAMIENTO EXTRACTIVO**

El presente registro se otorga para la conservación, manejo y aprovechamiento sustentable de la especie: ***Chiranthodendron pentadactylon* (árbol de manitas).** Asimismo se otorga en resguardo un ejemplar de la especie citada con marcaje etiqueta CHPE 1.

Previo a cualquier tipo de aprovechamiento que pretenda realizar de los ejemplares de flora silvestre existentes en la UMA registrada, debe contar con la aprobación del respectivo plan de manejo, estudios poblacionales y solicitarlo en el formato oficial.

Figure S1. Register management unit for the conservation of wildlife known by its acronym in Spanish UMA

MEDIO AMBIENTE

SECRETARÍA DE MEDIO AMBIENTE Y RECURSOS NATURALES

México 2021 Año de la Independencia

Delegación Federal de SEMARNAT
en el *Estado de México*
Subdelegación de Gestión para la
Protección Ambiental y Recursos Naturales

DFMARNAT/0706/2021
Toluca, México a 24 de febrero de 2021

A continuación, se indican las coordenadas UTM de la poligonal de la UMA **"FLOR MANITA"**.

VÉRTICE	UTM	
	X	Y
1	2130163.1	414594.68
2	2130181.26	414612.56
3	2130166.05	434631.3
4	2130146.53	434613.42
5	2131163.1	414594.68

ESTE REGISTRO QUEDA SUJETO AL CUMPLIMIENTO DE LAS CLÁUSULAS DE OPERACIÓN ANEXAS, CUALQUIER VIOLACIÓN O INCUMPLIMIENTO DARÁ ORIGEN A LA INSTAURACIÓN DE UN PROCEDIMIENTO ADMINISTRATIVO ANTE LA AUTORIDAD COMPETENTE PARA PROCEDER A LA CANCELACIÓN DEL REGISTRO Y LA APLICACIÓN DE LA NORMATIVIDAD CORRESPONDIENTE, SEGÚN SEA EL CASO.

El presente se emite en apego al principio de buena fe, al que se refiere el artículo 13 de la Ley Federal de Procedimiento Administrativo (LFPA), tomando por verídica la información presentada por el C. David Guajardo Ruz, en su escrito de fecha 18 de febrero del año en curso, en caso de existir falsedad en la información manifestada, el Promovente se hará acreedor a las sanciones correspondientes de acuerdo a lo dispuesto en las fracciones II, IV y V del artículo 420 Quáter del Código Penal Federal, referente a los delitos en materia ambiental.

ATENTAMENTE

SECRETARÍA DE MEDIO AMBIENTE
Y RECURSOS NATURALES
ESTADO DE MÉXICO

ING. JOSÉ ERNESTO MARÍN MERCADO
Con fundamento en lo dispuesto por el artículo 84 del Reglamento Interior de la Secretaría de Medio Ambiente y Recursos Naturales, en suplencia, por ausencia del Titular de la Delegación Federal de la SEMARNAT en el Estado de México[1], previa designación mediante oficio No. 01243 de fecha 28 de noviembre de 2018, firma el presente el *Subdelegado* de Gestión para la Protección Ambiental y Recursos Naturales

c.c.p.- Ing. Federico Ortiz Flores.- Encargado del despacho de la Delegación de la Procuraduría Federal de Protección al Ambiente en el Estado de México.- Toluca, Méx.

JEMM'MMH'RAAL **Bitácora: 15/U1-0402/02/21**

1 *En los términos del artículo 17 Bis en relación con los artículos Octavo y Décimo Tercero Transitorios del Decreto por el que se reforman, derogan diversas disposiciones de la ley Orgánica de la Administración Pública Federal, publicado en el Diario Oficial de la Federación el 30 de noviembre de 2018.*

Andador Valentín Gómez Farías No. 108, San Felipe Tlalmimilolpan. Toluca Estado de México, C.P. 50250.
Tel.: (722) 276 7835 y 276 7852 www.gob.mx/semarnat

Figure S1. (*continued*)

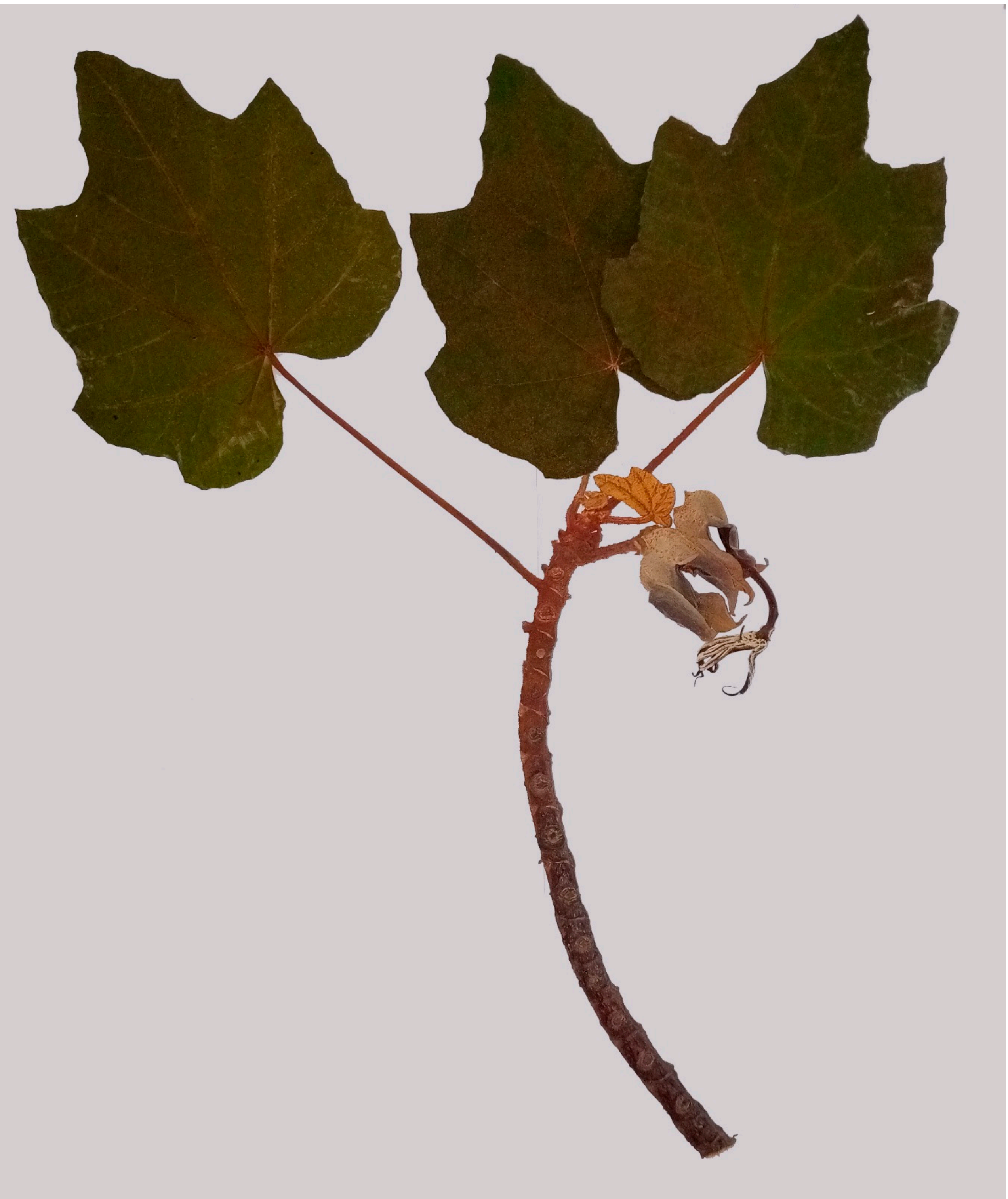

Figure S2. Herbarium specimen with the number 181307 collected in: Paseo Sta. Ana #142, San Carlos, Metepec, Edo de México.

Asunto: Constancia de ejemplar botánico.

A quien corresponda

PRESENTE

Por medio de este medio se hace constatar que el ejemplar de Herbario con el numero 181307 colectado en Paseo Sta Ana #142, San Carlos en Metepec, Edo de México, el día 22 de febrero del 2023, corresponde a Chiranthodendron pentadactylon, de la Unidad de Manejo para la Conservación de la Vida Silvestre (UMA) "Flor de Manita" lo asegura permiso SEMARNAT-UMA-IN-325-MEX/21.

Sin existir otro asunto que tratar, aprovecho para enviarle un cordial saludo.

"POR MI RAZA HABLARÁ EL ESPÍRITU"
Ciudad Universitaria a 30 de junio de 2023

Atentamente

Dr. Jaime Jiménez Ramírez
Herbario de la Facultad de Ciencias.
Universidad Nacional Autónoma de México

Figure S2. (*Continued*)
